# Remote Estimation of Blood Pressure Using Millimeter-Wave Frequency-Modulated Continuous-Wave Radar

**DOI:** 10.3390/s23146517

**Published:** 2023-07-19

**Authors:** Lovedeep Singh, Sungjin You, Byung Jang Jeong, Chiwan Koo, Youngwook Kim

**Affiliations:** 1 Department of Electrical and Computer Engineering, California State University, Fresno, CA 93740, USA; 2 Electronics and Telecommunications Research Institute, Daejeon 34129, Republic of Korea; 3 Department of Electronic Engineering, Hanbat National University, Daejeon 34158, Republic of Korea; 4 Department of Electronic Engineering, Sogang University, Seoul 04107, Republic of Korea

**Keywords:** blood pressure, pulse wave velocity, pulse transit time, pulse pressure, FMCW radar

## Abstract

This paper proposes to remotely estimate a human subject’s blood pressure using a millimeter-wave radar system. High blood pressure is a critical health threat that can lead to diseases including heart attacks, strokes, kidney disease, and vision loss. The commonest method of measuring blood pressure is based on a cuff that is contact-based, non-continuous, and cumbersome to wear. Continuous remote monitoring of blood pressure can facilitate early detection and treatment of heart disease. This paper investigates the possibility of using millimeter-wave frequency-modulated continuous-wave radar to measure the heart blood pressure by means of pulse wave velocity (PWV). PWV is known to be highly correlated with blood pressure, which can be measured by pulse transit time. We measured PWV using a two-millimeter wave radar focused on the subject’s chest and wrist. The measured time delay provided the PWV given the length from the chest to the wrist. In addition, we analyzed the measured radar signal from the wrist because the shape of the pulse wave purveyed information on blood pressure. We investigated the area under the curve (AUC) as a feature and found that AUC is strongly correlated with blood pressure. In the experiment, five human subjects were measured 50 times each after performing different activities intended to influence blood pressure. We used artificial neural networks to estimate systolic blood pressure (SBP) and diastolic blood pressure (SBP) with both PWV and AUC as inputs. The resulting root mean square errors of estimated blood pressure were 3.33 mmHg for SBP and 3.14 mmHg for DBP.

## 1. Introduction

Blood pressure is a parameter that provides fundamental yet critical information pertaining to human health. High blood pressure, also termed hypertension, is among the riskiest symptoms of heart disease. Hypertension is the leading cause of mortality around the world; moreover, approximately 30% of adults have the disease [1,2]. Hypertension occurs when blood vessels become narrower and stiffer, permanently increasing the pressure needed to force blood through the constricted arteries and veins. Similarly, low blood pressure or hypotension is also a sign of poor heart function, indicating that the heart may be at risk of failure. The commonest symptoms of hypotension are dizziness or headache; however, other symptoms such as lethargy and bradycardia (a slow pulse), constipation, visual disturbances, and nausea may also appear. Therefore, accurate measurement, as well as regular monitoring of blood pressure, are essential to detect signs of health disturbances in advance [3]. 

Blood pressure is a function of cardiac output, systemic vascular resistance, and arterial stiffness. It also varies depending on the individual’s psychological situation, emotional state, physical activity, and relative health/disease status. In general, blood pressure consists of systolic blood pressure (SBP) and diastolic blood pressure (DBP); in addition, the difference between SBP and DBP is called pulse pressure (PP). These pressures are measured in terms of millimeters of mercury (mmHg).

Historically, the cuff-based sphygmomanometer has been the most widely used instrument employed to measure blood pressure. A sphygmomanometer has many variations that can be used to perform measurements at different body locations, including the arm, wrist, and finger. In general, blood pressure is measured in the upper right arm by the inflation and deflation of a cuff, sometimes periodically repeated, which controls the flow of blood in the vessels. While this method represents the most direct way to measure blood pressure, it has several disadvantages. Examples include the time needed to take the reading through the process of inflation and deflation, the discrete and not continuous nature of the measurement, and the discomfort and inconvenience of wearing the cuff. 

Many alternative methods for blood pressure measurements have been suggested. Researchers have proposed several blood pressure measurement methods based on pulse wave velocity (PWV). The relationship between blood pressure and PWV has been studied extensively, ultimately revealing that PWV is proportional to blood pressure [4]. Therefore, PWV can be used to estimate SBP and DBP. In practice, PWV is measured by the pulse transit time (PTT) between two nodes in different parts of the body, which is inversely proportional to the PWV. A diverse dataset has been recorded and analyzed based on the PTT from electrocardiogram (ECG) and photoplethysmogram (PPG) measurements.

The use of only PPG has been advanced to measure blood pressure continuously and conveniently using wearable devices. PPG sensors have been installed in devices such as smartwatches and wrist bands that can provide blood flow information continuously at specific sites, providing a less intrusive measurement method. The device obtains the reflection of infrared waves from the movement of the blood vessel and analyzes the waveform to estimate blood pressure [5]. Scholars have studied diverse features, including area, width, peak, derivatives, and various delays, to identify correlations with SBP and DBP [6]. In [7], other morphological features of PPG, such as pulse amplitude, area under the curve (AUC), and width, were examined. According to the research findings, the SBP pressure was related more closely to the signal’s systolic peak that is the larger initial peak. These approaches have been adapted to the design of portable systems for continuous cuff-less blood pressure measurement, benefiting numerous people with pre-existing heart conditions. Furthermore, deep learning has been a critical development in analyzing PPG signals, facilitating the investigation of a large dataset [8]. The raw PPG, the first derivative of PPG, and the second derivative of PPG as well as spectral information were extracted for use as the input for deep neural networks. 

Recent progress has featured the use of optical imaging of facial patterns to calculate PPG information directly from facial variations depending on heartbeats [9]. However, facial optical data can greatly vary from one individual to another, as skin texture and lighting conditions can influence measurement readings. Another method that combined an ECG and radar was also evaluated to measure PTT [10]. However, because an ECG requires the placement of multiple electrodes on the chest to record the electrical signal of the heart, the method is not suitable for contactless blood pressure measurement. 

In [11], the paper successfully utilized Doppler radar to acquire chest displacement waveforms resulting from the expansion of the aorta. Subsequently, a correlation between chest displacement and pulse pressure was explored. Radar-based pulse transit time (PTT) measurement was conducted in [12], demonstrating its potential for estimating blood pressure. This particular study focused on measuring the time delay of pulses from the heart and wrist. In [13], researchers successfully captured the detailed shape of the pulse wave using radar technology. A comparison was made between waveform displacements measured with both radar systems and laser sensors. Building upon the analysis of pulse wave shapes in [14], blood pressure estimation was achieved. They measured three data samples from three human subjects, and the estimation error was reported to be 2.66 mmHg. Furthermore, employed deep learning techniques to transform waveform distortions caused by respiration and slight body movements into a clean pulse wave for PTT calculation [15]. However, it is important to note that the aforementioned research relies on the accurate acquisition of the pulse wave’s detailed shape, which can be challenging due to various factors such as the subject’s movement, clothing, and the aspect angle of the radar. 

In this paper, we investigate the feasibility of using frequency-modulated continuous-wave (FMCW) radars to achieve a contactless estimation of a human subject’s blood pressure without the detailed shape of the pulse wave. The FMCW radar has been used to detect sub-millimeter level vibrations of a body part remotely. Although Doppler radar has also been previously studied for the detection and remote sensing of various vital physiological variables such as respiration, heart rate, blood vessel motion, and pulse pressure [16,17,18], a technological method for continuous noninvasive remote blood pressure monitoring has remained elusive. We investigate the feasibility of a practical way to estimate the blood pressure using PTT and the simple characteristics of pulse waves. First, the PTT between the chest and the wrist was measured. Two radars were employed, directed at the chest and wrist of a human subject to detect skin displacement due to the blood pulse. The synchronized reading from the two radars was able to provide the travel time delay of the blood pulse between the chest and wrist, yielding the PWV, which was used to estimate blood pressure through a regression model. In addition, we suggested a method for estimating blood pressure using the waveform measured from only the wrist. Based on the strong correlation found between the PWV and the time history of the skin displacement, we analyzed the received pulse waveform to find a simple parameter related to blood pressure. Specifically, we investigated the AUC that represents the sharpness of peaks in the received wave as a primary feature to estimate blood pressure in case the detailed features are not well acquired. In the measurement campaign, five human subjects participated. Each subject was measured 50 times under different body conditions designed to vary the individual’s blood pressure. Then, the blood pressure was estimated using PTT and AUC using a regression model on the basis of polynomial approximation and artificial neural networks. The root mean square errors between the measured data and the model were calculated and compared. 

## 2. Background and Previous Methods for Measuring Blood Pressure

To date, the sphygmomanometer has been the gold standard for blood pressure measurement. When the cuff is fully inflated, the cuff’s high pressure completely blocks the artery from allowing any flow of blood. As the cuff pressure slowly deflates, the cuff pressure eases enough to let the artery start to resume the blood flow, which is SBP. Meanwhile, DBP is obtained when blood flow is normal, without any external pressure from the cuff, which causes no vibrations in the artery. Scholars have recognized PP as a more accurate diagnostic method than blood pressure by itself, especially in older adults. High PP is correlated with cardiovascular risk factors such as diabetes and smoking. 

Measurement of PP along with heart rate also enables the calculation of stroke volume and cardiac output. The PP, which equals the volume over compliance value, is directly proportional to the stroke volume and therefore to the displacement of the heart wall. Compliance is determined by the physical properties of the artery at a specific location. The aorta has the highest compliance in the arterial system due, in part, to a relatively greater proportion of elastin fibers versus smooth muscle and collagen. This characteristic serves the crucial function of damping the pulsatile output of the left ventricle, thereby reducing the initial systolic pulse pressure but slightly raising the subsequent diastolic phase. If the aorta becomes rigid because of disorders such as arteriosclerosis or atherosclerosis, the pulse pressure will be very high as the aorta becomes less compliant due to the formation of rigid lesions in the aorta wall.

**Figure 1:**
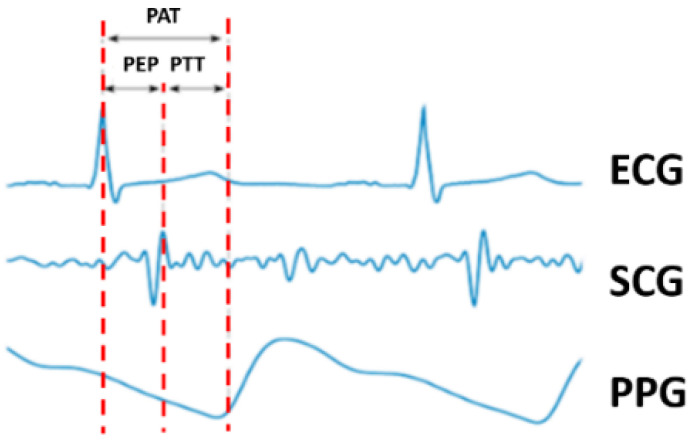
The relationship between PAT, PTT, and PEP.

Pulse arrival time (PAT) has also been recognized as having a strong correlation with blood pressure. The definition of PAT specifies the time elapsed between an ECG signal from the chest and the peak from the PPG signal measured at a second site. In other words, this time delay is the sum of the pulse ejection period (PEP) and PTT. The PEP is obtained from the ECG and SCG. PAT is inversely proportional to blood pressure, thus providing a method to measure blood pressure. Alternatively, PTT-based blood pressure measurements that have also been explored have set the PEP, which is the period from ventricular depolarization to ventricular ejection, as a constant value [19,20]. However, another study reported that PEP could vary due to breathing variations and premature contraction of the heart muscle [21]. Hence, a PTT-based method offers a better solution as it does not include the PEP delay. Figure 1 depicts the relationship between these variables. Notably, although some previous studies have used PAT and PTT without any alteration, such approaches were incorrect as the two phenomena represent different delays.

Numerous equations have been proposed to describe the correlation between blood pressure and various parameters, such as PAT, PTT, PEP, and PWV. The most popular equations describing the relationship between PWV and blood pressure are the Moens–Korteweg (M.K.) equation and Hughes equation [22,23]. The equations can be described as follows:

(1) $$M.K.\;\mathrm{Equation}:\;PWV=\frac{L}{PTT}=\sqrt{\frac{E\cdot h_{o}}{2\rho \cdot R_{o}}}$$

(2) $$\mathrm{Hughes}\;\mathrm{Equation}:\;E=E_{o}\cdot exp(\varsigma P)$$

where *L* denotes the vessel length, *ρ* represents the blood density, *R_o_* is the inner radius of the vessel, *h_o_* signifies the vessel wall thickness, ζ is a constant, *E_o_* is the zero-pressure modulus of the vessel wall, and *P* refers to the blood pressure within the vessel. Based on these two equations, blood pressure can be computed from the PTT, assuming all other parameters are held constant. In [24], a relationship between PWV and blood pressure was derived and proven through practical results. The main goal of the paper was to establish an equation without assumptions made in the M.K. and Hughes equations that required multiple inputs, including elastic modulus, artery radius and thickness, blood density, and material coefficients. The authors argued that using their relation between PWV and blood pressure, as shown in Equation (3), provided more accurate results than previous methods.

(3) $$BP=a\cdot PWV^{2}+b$$

The constant *a* is related to elastic modulus at zero pressure, and *b* is an overall offset to the entire curve. Meanwhile, a PTT-based blood pressure calculation was made in [25], as follows:

(4) $$BP=\frac{K_{1}}{PTT}+K_{2}$$

This equation features the inverse relationship between PTT and blood pressure. The two constant values are subject-dependent, which are related to the elastic modulus, distance to the wrist, blood density, and other factors. It is found that Equation (4) has a strong correlation with DBP [19].

## 3. Radar Measurement

PTT has previously been measured using contact-based devices. In contrast, this project focused on obtaining the PTT parameter with no contact using millimeter-wave FMCW radar. The time delay of the pulse on two parts of the body (e.g., the chest and wrist) can provide the PTT, which can be simply described as the time delay of the pulse measured at two locations. Blood flow causes the chest vibration and wrist artery pulse transit to make a sudden outward push of the skin. This skin displacement yields the change of phase of the received radar signal. Our study employed measurement at a subject’s chest and wrist to determine the time delay of the pulse. Even though this study focused on PTT between the heart and wrist because the skin vibration could be easily detected due to the strong pulse, other locations, including the foot, ankle, knee, and elbow, would also be usable. The PTT values were used to correlate with the DBP based on the relation between the two parameters revealed in Equation (4). 

Two AWR1243 FMCW radars developed by Texas Instruments were employed to obtain the degree of skin displacement at the chest and wrist through phase variation. The FMCW radar used in the current study operated at 77 GHz and provided a maximum of 4 GHz of bandwidth. The vital sign measurements were taken at a distance of 0.5 m. Table 1 displays the parameters used for data capture. The chest radar operated at 77 GHz, while the wrist radar operated at 79 GHz to avoid interference. Other than the operating frequency, all other parameters were the same for both radars. The two radars were synchronized in time to measure the delay. For this research, IRB approval was obtained from California State University, Fresno (IRB-21-ECE00), and all participants completed the required IRB consent form for human data capture.

**Figure 2:**
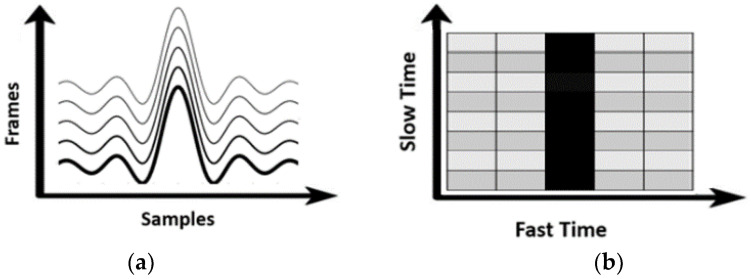
(**a**) The range profile with frame (time), and (**b**) range–Doppler map.

We conducted the fast Fourier transform to the time domain data (fast time) with each frame to construct a range–time map in the form of a as shown in Figure 2a. Then, the next step entailed locating the range bin where the human subject was detected. Even though the identified range bin is constant (Figure 2b) as the human subject was stationary, the phase of the signal varied due to the small displacement of human skin. The frame had a periodicity of 0.5 ms, which provided sufficient accuracy in measuring time delay at the sub-millisecond level, reflecting the range of PTT values in terms of a few hundred milliseconds around the body.

The phase information obtained from the range profile was subject to DC noise. As a high DC component could have negatively influenced the phase accuracy required to obtain a small displacement, this noise was removed by compensating for the error. Among various methods [12,13], the least square method was chosen to obtain the shift of data points. After removing the DC noise, complex data of the maximum magnitude in the range profile per frame were used to obtain phase information using arctangent demodulation, which provided the displacement with time. The phase unwrapping was performed on the phase vector to remove a discontinuity [12]. 

**Figure 3:**
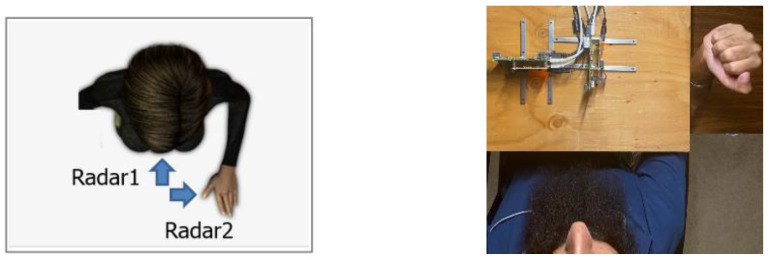
Measurement setup for two radar devices.

In the measurement campaigns, as shown in Figure 3, the radars were placed facing the chest and wrist. The distance from each radar device to the chest and wrist was 0.5 m.

**Figure 4:**
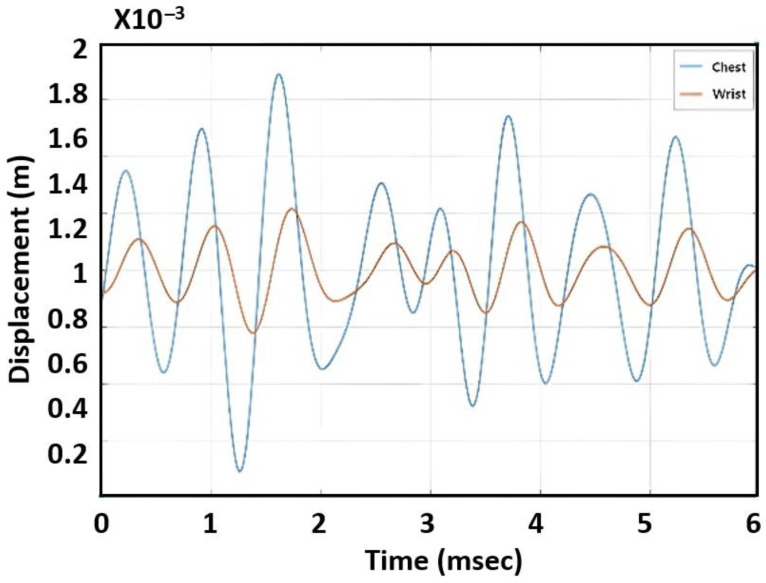
Measured displacement from chest (blue) and wrist (red).

Figure 4 shows displacement variations for a subject’s chest and wrist measured by radar. Because the blood pulse traveled from the chest to the wrist, it was observed that the phase of the chest signal led the one recorded from the wrist. The PTT was found by calculating the time delay between the two waveforms. In this measurement, PTT was around 210 ms. By comparison to PPG sensors that can obtain detailed systolic and diastolic peaks [6,7], our data from using radar only show the systolic peak while the diastolic phase could not be measured accurately.

## 4. PTT Measurements

As shown in the previous section, the proposed radar system was able to measure the PTT. To determine the relationship between the PTT and blood pressure, it is required to take the blood pressure measurement using a cuff for multiple participants. We used Omron’s Sphygmomanometer (HEM-FL31), and the cuff readings were performed on the left upper arm. The measurements were taken while the participating individual sat on a chair. 

The five individuals who participated in the investigation ranged in age from 20 to 76 years and included both genders. The characteristics of the five individuals can be found in Table 2. In the table, arm length was defined as the length from the heart to the wrist. Notably, the third participant was taking medication on a regular basis to regulate hypertension and diabetes. The remaining participants were healthy and did not take any medicine at the time of the investigation. Each subject was measured 50 times under different body conditions to collect diverse blood pressure readings. As each individual’s blood pressure is a function of the physical and psychological status, we conducted blood pressure measurements under the following conditions to have diverse blood pressure ranges: upon awakening in the morning; after breakfast; after vigorous activities such as running, exercising, pushups, and running upstairs; and before going to bed. All measurements were taken using the same measurement setup. It took 2–3 days per person to obtain 50 readings. Our data collection process yielded a total of 250 data samples.

**Figure 5:**
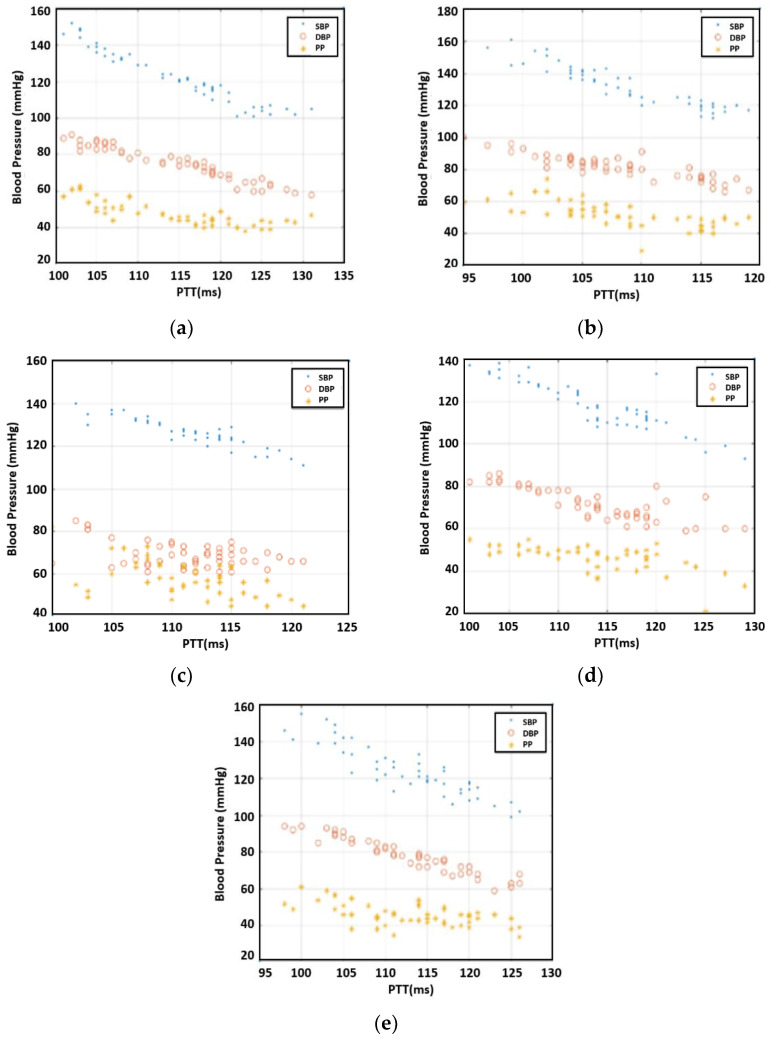
The relationship between blood pressure and PTT, (**a**) subject 1, (**b**) subject 2, (**c**) subject 3, (**d**) subject 4, and (**e**) subject 5.

After the measurements, we investigated the correlation between PTT and SBP/DBP/PP for each individual. Figure 5 offers the results for 50 readings per participant. As expected, the inverse relationship between PTT and SBP/DBP/PP was observable. For the case of Individual 3, DBP varied greatly compared to the other four individuals, resulting in a large variation in PP, as well. Overall, the correlation between PTT and DBP was more pronounced than that of PTT with SBP and PP.

**Figure 6:**
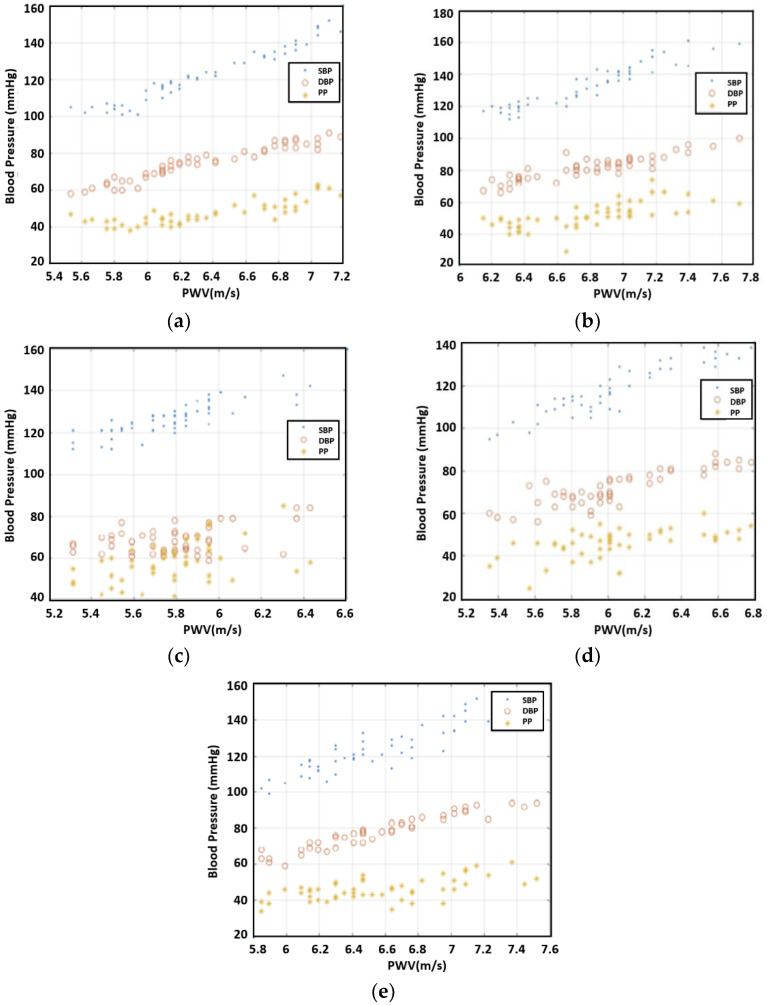
The relationship between blood pressure and PWV, (**a**) subject 1, (**b**) subject 2, (**c**) subject 3, (**d**) subject 4, and (**e**) subject 5.

Even though the PTT was related to blood pressure, PWV was more directly related as people’s arm length varied. The PTT divided by arm length provided the PWV. The values that we calculated for PWV are presented in Figure 6. The figure shows a trend that PWW is proportional to SBP/DBP/PP.

**Figure 7:**
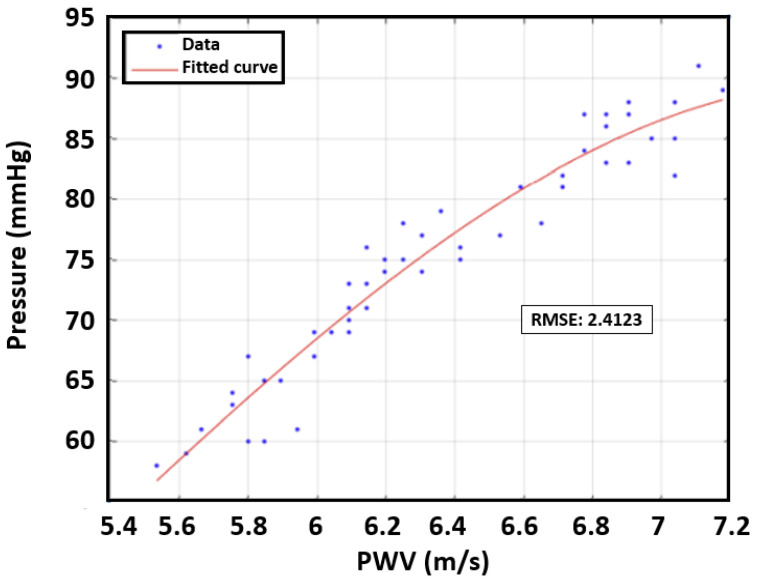
The relationship between DBP and PTT for the first participant.

In the next step, we evaluated the error for each case. Polynomial curve fitting was obtained by using MATLAB to determine the best polynomial regression model. The root mean square error (RMSE) was used to evaluate this case as it quantified the error between the model and measured data. The third-order polynomial was found to provide the best RMSE results for the preliminary data. Figure 7 presents an example of DBP depending on PWV with curve fitting results for the first human subject. As seen in Table 3, the PTT revealed the strongest relationship with DBP as the RMSE was 3.69 mmHg.

Notably, Individual 3, with a pre-existing condition, had the most uniquely different dataset, especially the DBP values, which provided the largest RMSE. This outcome demonstrates that the measurements can be tailored for individuals with specific backgrounds. Individual 2, who also had a case of hypertension, yielded average pressures that were much higher than other individuals’ normal readings. Nevertheless, this individual’s dataset was able to fit much better compared to that of Individual 3. Without Individual 3′s data, the average RMSE for DBP is 3.23 mmHg.

## 5. Area under the Curve (AUC) Measurement and Analysis

This section describes our investigation of the feasibility of estimating blood pressure with a single radar. The previous approach using PWV required two radar devices and arm length information, which added complexity as well as increased the possibility of error. In this step, we focused on finding the correlation between blood pressure and the characteristic of the displacement graph shown in Figure 4. Our rationale was that the PWV would affect the shape of the graph—in particular, the sharpness of the peak in the graph. If the PWV increased, the peak would be sharper because the time response from the skin due to the heart pulse would be faster. We tested various features to evaluate the shape of the peak, including the variation of derivatives (first derivative, second derivative, third derivative), peak width, and AUCs. Among these features, the AUC demonstrated the best correlation. 

**Figure 8:**
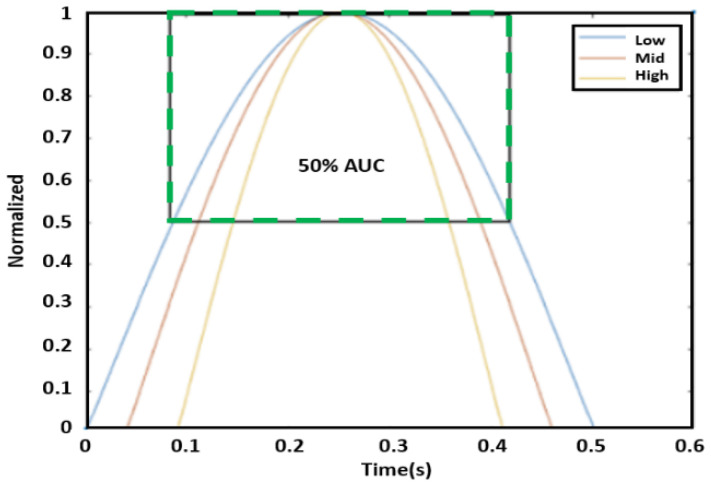
AUC measurement at the level of 50% from a peak.

**Figure 9:**
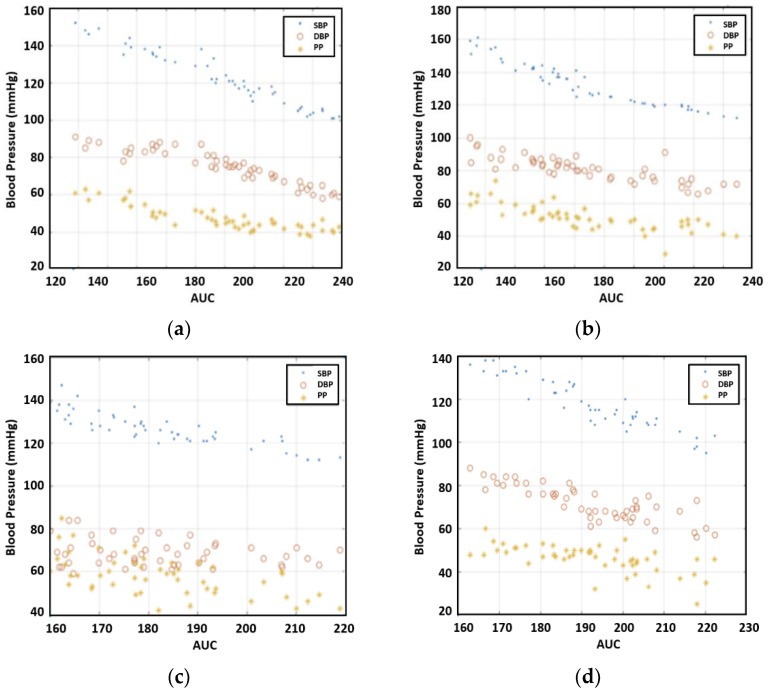

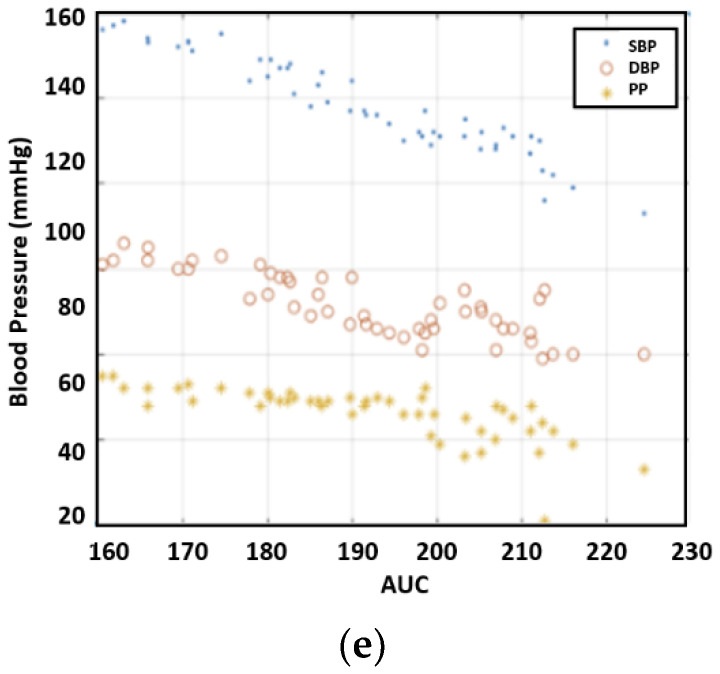
Individuals’ AUC vs. blood pressure, (**a**) subject 1, (**b**) subject 2, (**c**) subject 3, (**d**) subject 4, and (**e**) subject 5.

AUC is intended to measure the sharpness of the peak. First, the amplitude of each peak of the displacement graph was normalized to the unit level because the peaks of the phase graph varied. After normalizing amplitude to one, we defined AUC as the area bounded by the graph itself and a line of 50% of the peak (i.e., 0.5), as shown in the green box of Figure 8. Three peaks from the 10 s data were selected to find an average AUC value for each reading. In particular, the three highest peaks were extracted for this process. Figure 9 illustrates the correlation between blood pressure and AUC. The experimental results uncovered a strong correlation between blood pressure readings and AUC at half amplitude. Furthermore, on average, AUC measurements provided a closer correlation with SBP compared to PTT.

**Figure 10:**
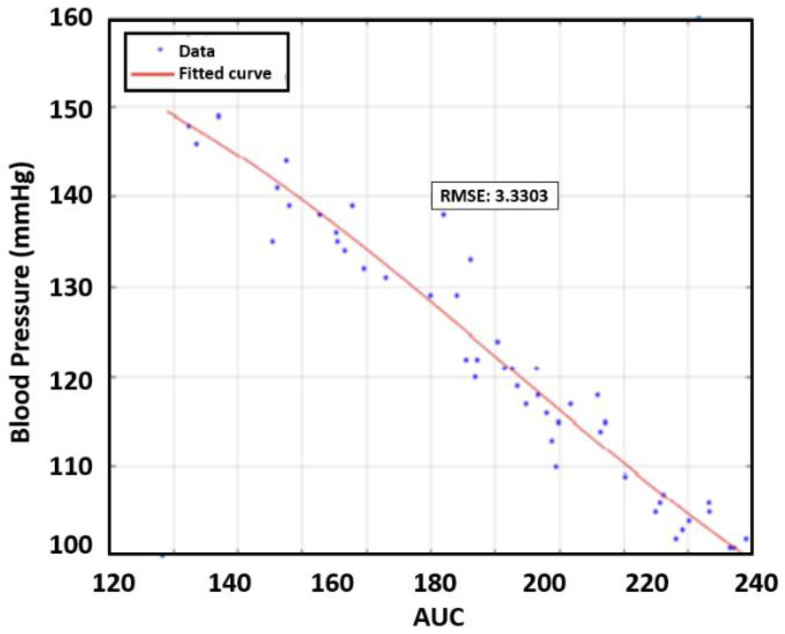
Example RMSE for AUC vs. SBP for Individual 1.

To evaluate the AUC, we introduced a regression model that found the best fit to the data to determine the RMSE as presented in Figure 10. As can be seen in Table 4, the AUC displayed the strongest relationship with SBP. In contrast to the average RMSE value for PTT versus DBP, which was 3.69, the value for AUC versus SBP was 3.60. Without Individual 3′ data, the RMSE for SBP decreases to 3.49 mmHg. 

## 6. Correlation between Combination of Features vs. DBP/SBP for Multiple People

**Figure 11:**
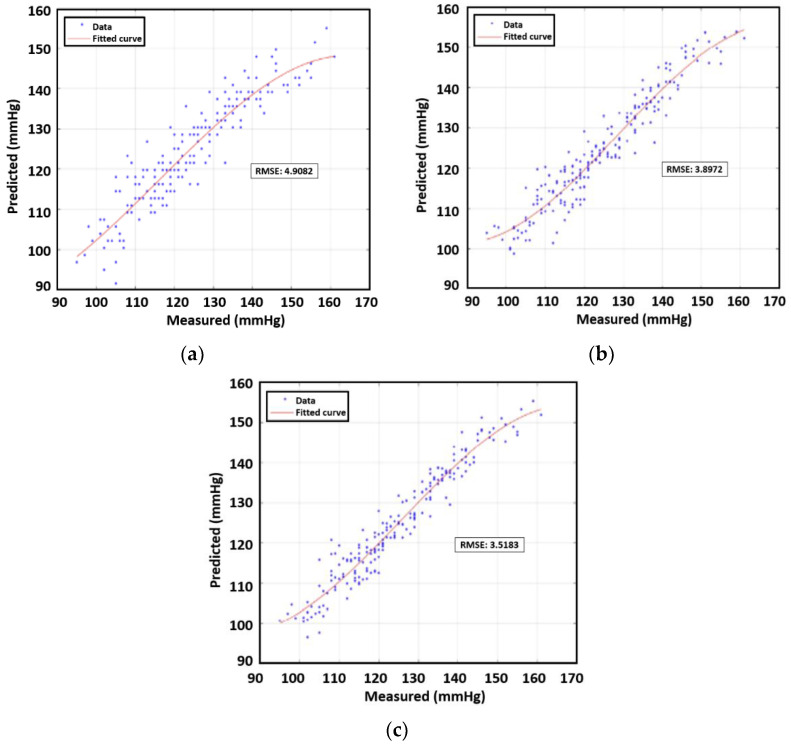
RMSE using polynomial for SBP using (**a**) PWV, (**b**) AUC, and (**c**) PVW+AUC for four participants.

**Figure 12:**
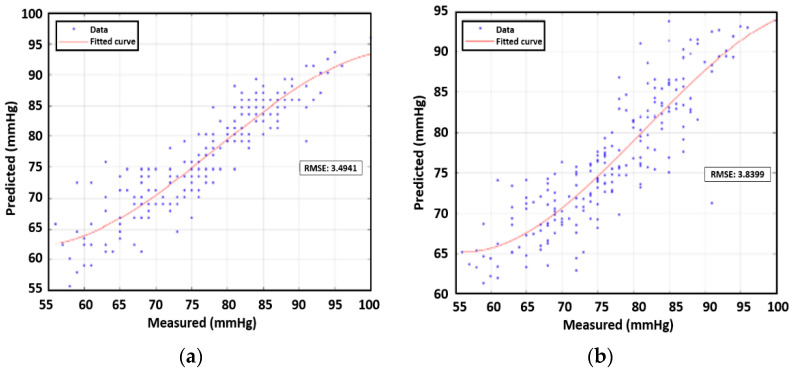

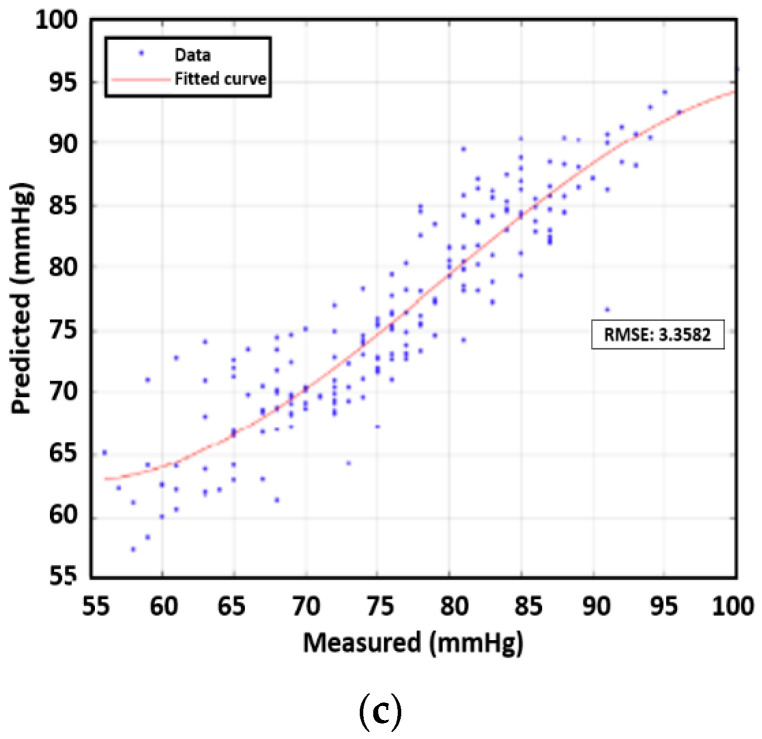
RMSE using polynomial for DBP using (**a**) PWV, (**b**) AUC, and (**c**) PVW+AUC for four participants.

We investigate the feasibility of estimating blood pressure using both PWV and AUC for data samples from multiple subjects. All data points except those for subject 3 were used in prediction analysis with machine learning. Subject 3′s data were an outlier case due to medication. First, we constructed third-order polynomial regression models. The next step entailed plotting the true values and the predicted value from the regression model. RMSEs were found for the four remaining human subjects’ data, which were analogous to the training error in the context of machine learning. On the basis of the calculations, for single feature correlation, SBP and DBP were both better correlated with AUC than PWV. The RMSE of SBP was 3.89 mmHg while that of DBP was 3.83 mmHg. In addition, we applied a multivariate polynomial regression model using two inputs of PWV and AUT, which yielded values of 3.51 mmHg for SBP and 3.35 mmHg for DBP, as shown in Table 5 and Table 6. The polynomial approximation for SBP and DBP are illustrated in Figure 11 and Figure 12 respectively, showcasing the correlation between measured and predicted values.

Next, we used artificial neural networks (ANNs), a popular machine learning technique, to predict blood pressure given PWV and AUC. ANNs are made of several layers; each neuron has a weight and activation function that calculates outputs for the following layer. In this study, the input layer took the features calculated and passed them down to the hidden layers. The fully connected layers were used for the hidden layers. The hyperparameter of the ANN was heuristically determined. The model consisted of two fully connected layers of which the sizes are 20 and 10, respectively. The architecture was kept small due to the small amount of available data. It is noted that a deeper neural network was applied, but it caused an overfitting problem that resulted in a high test error. For training the net, we used the Broyden–Fletcher–Goldfarb–Shanno quasi-Newton algorithm as a loss function minimizer. A fivefold cross-validation was also performed to ensure that the model did not overfit due to the small amount of data. Cross-validation allowed the model to be trained and validated based on different data for each iteration. Then, the model that provided the lowest loss was used on a test set of 25 data points. The training and validation data consisted of 175 data points. Table 5 and Table 6 present the test errors from the ANN procedure.

The results reveal that AUC was the most important feature for estimating SBP, which was in line with the conclusion for individual cases. The use of a combination of features decreased the estimation error. For DBP, the PWV was a key feature; here, the use of combination features was also able to decrease the estimation error. Interestingly, the use of ANN performed better compared to the regression model.

## 7. Conclusions

This study proposed a method of contactless, continuous blood pressure estimation using millimeter-wave FMCW radar and reported preliminary results. The PWV and AUC data obtained from measurements from multiple human subjects were found to be strongly correlated with SBP and DBP. The lower RMSE value of SBP was from AUC while the combination of features even decreased the error. Regarding DBP, PWV was more correlated compared to AUC while the combination of features showed better results. However, Individual 3 who takes medicines for hypertension and diabetes, as an outlier, greatly increased the RMSE value for DBP.

This method can provide continuous monitoring of patients with zero contact, which is potentially useful in the medical field or in daily life, ensuring the subject’s safety and comfort while making the measurement unobtrusive or even invisible. For example, the proposed method using radar could be installed at home for individuals who might require continuous remote monitoring to detect any signs of a blood pressure-related disease. Therefore, the method can be used to be as an auxiliary method to the traditional methods, offers ease of use, and will become financially affordable as the price for radar decreases over time. Future studies can focus on the further development of remote monitoring systems for patients that can alert medical officials when blood pressure readings diverge appreciably from normal ranges. This remote contactless blood pressure measurement might increase the frequency of blood pressure readings, which can construct big data regarding blood pressure.

While these preliminary findings are promising, many obstacles remain to obtain more reliable results. Motion noise significantly affects the shape of the signal, so developments in signal processing techniques should be made to resolve the motion noise issue. While signals from ECG and PPG have many more details that can be exploited for blood pressure estimation, features from the radar are rather simple. Further research on feature extraction should be conducted. In addition, the pulse from the wrist is relatively weak. To capture the pulse from the wrist effectively, the use of terahertz radar needs to be considered.

## Figures and Tables

**Table 1:** Radar Parameters.

Parameter	Value
Operating Frequency	77 GHz/79 GHz
Chirp Slope	29.99 MHz/μs
ADC Sample Rate	2.5 MHz
Pulse Period	0.5 ms
Frames	20,000
Pulse Duration	57 μs
ADC Samples	128
Measurement Length	10 s

**Table 2:** Individual information.

Person	Age	Gender	BMI	Arm Length (m)	Note
1	20	M	19.4	0.725	Healthy
2	57	M	24.4	0.732	Hypertension
3	76	F	24.9	0.643	Hypertension and diabetes (Taking medication)
4	49	F	26.1	0.685	Healthy
5	24	M	20.6	0.737	Healthy

**Table 3:** Curve fitting RMSE using third-order polynomial for PWV.

Individual	SBP (mmHg)	DBP (mmHg)	PP (mmHg)
1	3.2172	2.4123	3.2489
2	4.3787	3.3941	5.5169
3	4.2366	5.5093	8.1687
4	4.8503	4.3938	5.4078
5	6.1266	2.7464	5.0139
Average	4.5619	3.6912	5.4712

**Table 4:** Curve fitting RMSE using third-order polynomial with AUC.

Individual	SBP (mmHg)	DBP (mmHg)	PP (mmHg)
1	3.3303	2.9734	2.8911
2	3.2498	4.5015	4.9076
3	3.8701	6.2428	7.6907
4	4.0904	4.5623	5.0837
5	3.4625	3.6507	3.8672
Average	3.6006	4.3861	4.8881

**Table 5:** RMSE using polynomial and ANN for SBP.

Features	SBP Using Polynomial (mmHg)	SBP with ANN (mmHg)
PWV	4.9082	5.7299
AUC	3.8972	3.4125
PWV+AUC	3.5183	3.33086

**Table 6:** RMSE using polynomial and ANN for DBP.

Features	DBP Using Polynomial (mmHg)	DBP with ANN (mmHg)
PWV	3.4941	3.1511
AUC	3.8399	3.2149
PWV+AUC	3.3582	3.1402

## Data Availability

Data sharing is not applicable due to human subjects’ privacy.
